# College students’ reception of social networking tools for learning in India: an extended UTAUT model

**DOI:** 10.1186/s40561-021-00164-9

**Published:** 2021-09-19

**Authors:** Irum Alvi

**Affiliations:** Department of Humanities, English and Applied Sciences (HEAS), Rajasthan Technical University, Kota, Rajasthan 324010 India

**Keywords:** Social networking tools, College students, Higher education, Intrinsic values, UTAUT

## Abstract

The term Social Networking Tools is used for social media applications accessible via mobile devices/smartphones; their use has become ubiquitous among college students, especially after the COVID 19 Pandemic, due to which the institutes of Higher education were shut down. A research gap was identified as the students’ acceptance of these learning tools has not been studied to the best of the author’s information, in India. The current study employs the conceptual model based on the UTAUT model by Venkatesh et al. (MIS Q 27(3):425–478, 2003), extended and modified by Khechine et al. (Br J Educ Technol 51 2306–2325, 2020. 10.1111/bjet.12905). The sample comprised 305 students, 48 females (15.7%) and 257 males (84.3%), with an average age of 18 years. Partial least squares structural equation modeling (PLS-SEM), a methodology of structural equation modeling which allows the assessment of any complex cause-effect model comprising latent variables was used for assessing the hypothesized model using SmartPLS version 3.2.9. The results show that the college students were impacted by Performance Expectancy PE, Effort expectancy EE, and Social Influence SI in shaping their behavioral intention BIU; Facilitating conditions FC and Intrinsic Values IV didn’t influence their behavioral intention. However, their behavioral intention BIU and their Intrinsic Values (IV) influenced their intention to use (IU) social networking tools for learning for Higher education, in the Indian context.

## Introduction

The use of Social Networking Tools for learning has become ubiquitous among college students. These are also termed as social networking sites, social networking communications, or Web 2.0, etc. College Students extensively use these emerging tools (Lim et al., 2014), for cross-cultural communications, knowledge acquisition, and self-examination (Yu et al., 2010). Social networking tools play a crucial part in the modernization of learning methodologies by making diverse and varied means to interact, entertain, interconnect, and correlate accessible to the students and teachers, and by paving the way towards a student-centered learning methodology, based on interaction rather than teacher-centered methodology, grounded on traditional pedagogical methodologies (Tapscott, 2008). The tools function as an important source for interaction as well as cooperation amongst learners (Al-Khalifa & Garcia, 2013).

Sites for social bookmark including blogs, wikis, etc.; SNS sites (LinkedIn, Facebook); sites for social contents (YouTube), networks for social communication (Skype, Google Hangout, etc.), and virtual reality applications (Second Life) are examples of such tools (Vaughan et al., 2013). Web 2.0 technology or Social media are commonly utilized for attaining successful joint learning interactions (Vaughan et al., 2013). Their utilization has increased tremendously for creating the social learning atmosphere, after the COVID 19 pandemic due to the lockdown imposed which limited social activities (Laato et al., 2020). However, despite the widespread use of such tools during the COVID 19 Pandemic in institutes of Higher education, there is a paucity of studies on students’ reception and acceptance of social networking tools (Khan et al., 2021). The tools enabled students and faculty to interact while observing social distance measures (Vordos et al., 2020) and enhanced connectivity and collaborative learning (Islam et al., 2020).

Since scholarly writings consider reception and acceptance as the sine qua non to accomplishment and productivity of technology, analyzing the determinant of adoption that incorporates social learning tools is significant in today's times (Taylor & Todd, 1995). Moreover, recognizing the determining factor will allow for the creation of effective technology implementation guidelines and the validation of investments (Davis, 1989). The present study aims to provide an enhanced appreciation of the level of students’ reception of such technology-based tools and their predictors, which may enable the responsible stakeholders, including administration, faculty, staff, and policymakers, to intervene and enhance the appeal of the tools/technologies. Prior studies have established that a student is less likely to accept technology-based learning if he believes they have little or no value for him (Crompton & Burke, 2018). The present study’s main objective is to identify the determining factors for college students’ intent to use (IU) social networking tools for learning in the Indian context, which has not been studied till date, to the best of the author’s information. Moreover, the study explores the influence of IV on college students’ behavioral intention (BIU) and intent to use (IU) social networking tools.

### Literature review and hypotheses

#### Social networking tools for learning

Social media comprises a wide range of web and mobile platforms that facilitate connecting and communicating among individuals in a virtual network (Naslund et al., 2020), using the multifaceted online setting (Al-Aufi & Crystal, 2015). College students frequently use them for accessing online teaching materials and secondary material, handling group tasks, and communicating with teachers (Anshari et al., 2017). These learning tools help and improve the learning process at any time and place, thereby constituting a progressing learning technology-based feature at various educational stages (Nikolopoulou, 2020). Hence, the term Social Networking Tools is used for social media applications accessible via mobile devices/smartphones, etc., such as Pinterest, Instagram, Facebook, Twitter, etc. (Yang, 2017). It also refers to the rapidly growing trend of using social media apps on mobile devices/computers/laptops, etc.; these tools use social software or/ and social media to promote education and the system for learning purposes (Pappas, 2012). They provide a real learning experience to the students, which helps in enhancing gratification, knowledge, and learning among them (Popescu & Cioiu, 2011). The aim of utilizing these tools is to make education easily accessible and widely available to all. It permits for students and/or technology interaction, which adds a new dimension to learning. Students learn through distance learning platforms such as Facebook, Twitter, etc. In informal/formal learning atmospheres, these tools assist in building relations between students, teachers, and learning content/material, thereby forming and enhancing active learning networks (Pappas, 2012).

The development of social networking tools in the field of education is accelerating quickly. The widespread use of such tools in education necessitates that teachers and students comprehend and adapt them for the successful implementation of education plans and policies and deployment of course content on e-platforms (Bai et al., 2021). Social media tools with their new and novel features provide opportunities for two-way communication, collaborative learning, feedback and assessment. Additionally, their continuous usage improves students' interaction and engagement, as the mode of interaction empowers the students to team up as well as interconnect overcoming geographical limitations; as such, it improves their academic performances (Berkani, 2020) and encourages teamwork, and provides a forum for them to deliberate upon new and novel ideas (Tess, 2013). The tools make feedback possible (Rahman et al., 2019), and are potent educational tools that enhance teaching and learning (Al-Bahrani et al., 2015). The adoption of social media tools in education has been studied in a variety of circumstances. However, only a few studies have deliberated students' adoption of social media for learning in higher education; as such, the study fills the research gap in extant literature.

#### Technology acceptance models for learning

Acceptance of technology-based learning is a hot topic in research nowadays (Cheng et al., 2020; Kumar & Chand, 2019). Several models have been used over the past years by researchers to investigate the acceptance of technology among students (Kumar & Bervell, 2019); these include the Technology Acceptance Model (TAM) (Davis, 1989), Model of Behavior (Davis et al., 1992), planned behavior theory (TPB) (Ajzen, 1991), Decomposed Theory of Planned Behavior (Taylor & Todd, 1995), personal computer use model (Thompson et al., 1991), diffusion of innovation theory (Rogers, 1995), and social cognitive theory (Compeau & Higgins, 1995). With the UTAUT model (Venkatesh et al., 2003), researchers were able to get an accurate extrapolation of users’ intent (Khechine et al., 2016). As such, the model enjoys good reliability and has been used extensively by several researchers for providing analytical understandings for the adoption of technology in new contexts (Venkatesh et al., 2016). However, the UTAUT model (Venkatesh et al., 2012) has been used infrequently in the context of higher education (Arain et al., 2019), although it is suitable for higher education. The present study uses the model, considering its fitness for the condition rather than its frequency of use. Furthermore, it is relevant to the context of learning using new technologies (Venkataraman & Ramasamy, 2018) and has been extended to social learning (Khechine & Augier, 2019). The present study further extends it to encompass social networking tools for learning in Higher education.

This study employs the UTAUT as an initial point and expands the originally offered model with the addition of one pertinent construct considered as significant in extant research on social networking tools for learning and their acceptance among the students i.e. Intrinsic Values (IV). Intrinsic value is considered as an affirmative and desired phenomenon to sustain the user's commitment and self-inspiration for utilizing any new technology (Turel & Serenko, 2012). It is described as the feeling of both pleasure and interest (Chiu & Wang, 2008) one feels while performing any task. Although users are interested in utilizing social media for individual reasons, its use in the academic environment isn’t guaranteed (Quong et al., 2018). Although the UTAUT model was expanded with the hedonic motivation construct (Nikolopoulou et al., 2020; Venkatesh et al., 2012), it emphasizes solely the pleasure dimension found by using it (UTAUT2). However, in the context of education, enjoyment and interest must also be taken into account as essential intrinsic drivers of acceptance and successful usage. As a result, it is suggested that the hedonic motivation construct from UTAUT-2 should be expanded by including two dimensions—enjoyment and interest. The IV variable, which is used to predict the adoption of social networking tools for learning in this study, includes both interest dimensions and enjoyment. Though the original UTAUT model was successfully used in other frameworks, the present study applied IV in the context of utilizing social networking tools for learning.

**Fig. 1:**
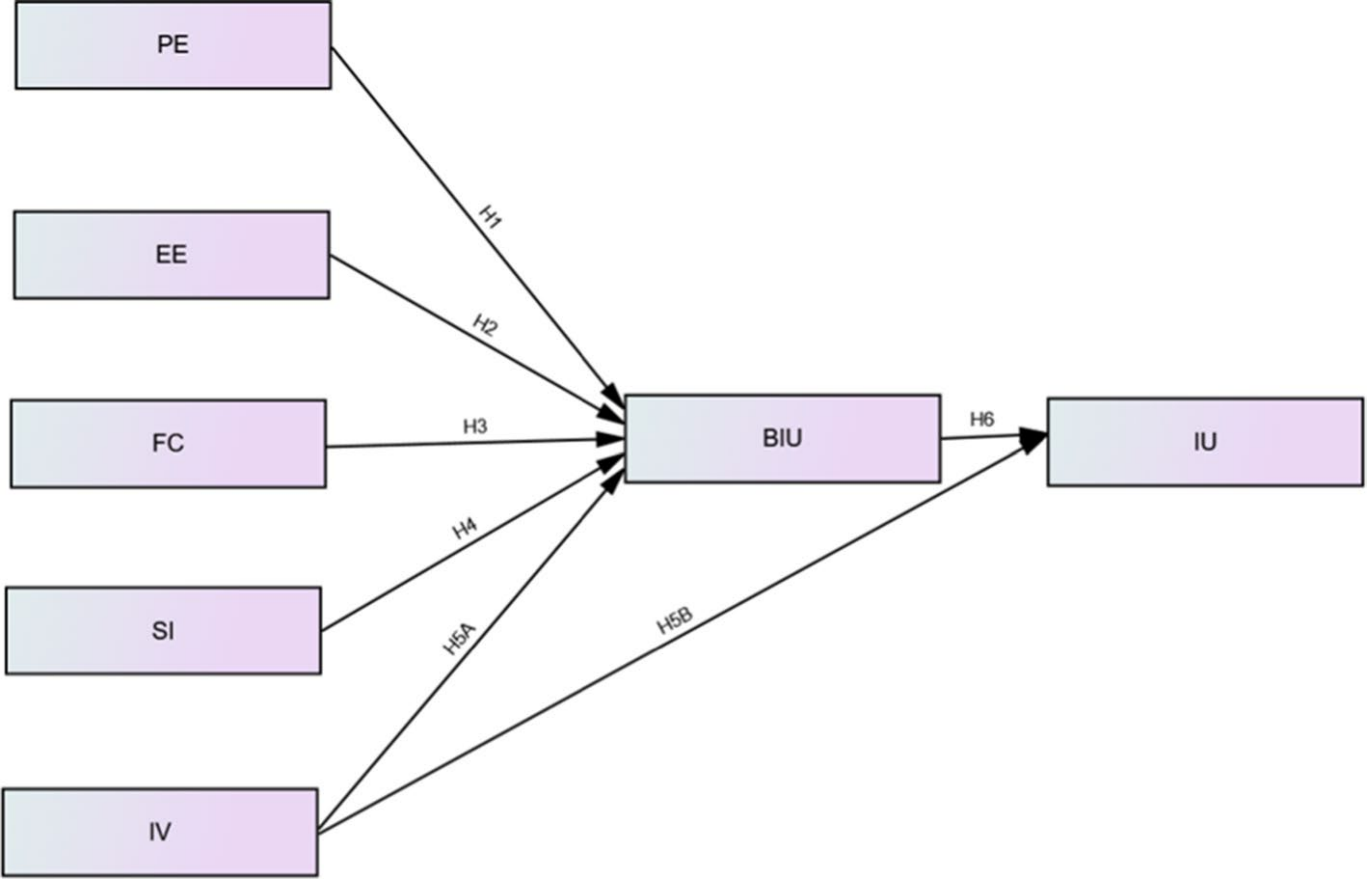
Conceptual model

Figure 1 shows the conceptual model that illustrates the hypothesized relationships between the select constructs for the reception of social networking tools for learning among college students. It is based on the original UTAUT (Venkatesh et al., 2003) which was extended by Khechine et al. (2020).

### Performance expectancy PE

The extent to which a person assumes utilizing the technology can aid him in improving performance is referred to as Performance Expectancy. The importance of affirmative relation between performance expectation and BIU/IU has been established in several prior studies: PE is considered as the best predictor of intent (Venkatesh et al., 2003). Performance expectation appears in several other models; Motivational Model with the construct named Extrinsic motivation, TAM and TAM 2 with the construct named Perceived utility, creativity diffusion theory with the construct named relative advantage, personal computer use with the construct named role fit, and the social cognitive theory with the construct named outcome perceptions have all proven the role played by PE. The relation between PE and BIU was established to be substantially affirmative (Venkatesh et al., 2016); as such, it is anticipated that PE will play a significant role in predicting the reception of social networking tools for learning among college students in India:

#### **H1**

PE has an affirmative significant influence on students’ BIU towards using social networking tools for learning.

### Effort expectancy EE

The extent of the ease with which technology can be used is referred to as Effort Expectancy. Other models' constructs for effort expectancy include Perceived Ease/Effort, and Ease of use. Venkatesh et al. (2003) maintain that EE is important merely throughout the initial phases of use which later fades away over time with the continuous usage of technology. Several empirical studies consider EE as essential for the reception of new tools and technologies (Wang & Wang, 2010). As a result, the following hypothesis was formulated:

#### **H2**

EE has an affirmative significant influence on students’ BIU towards using social networking tools for learning.

### Facilitating conditions FC

FC is characterized as a student’s belief in the existence of an organizational/technological set up to facilitate the utilization of technology-based tools for learning. Models like the Innovation Diffusion Theory, Planned Behavior Theory, Personal Computer Use Model, etc. all assimilate the notion of FC. Conditions that make it easier to use technology have an affirmative impact on real use (Venkatesh et al., 2003). However, when it came to predicting intent (Venkatesh et al., 2003) FC was found to add no value to PE or EE. Later studies (Khechine et al., 2016, etc.) indicated that a significant and positive relationship existed Between FC and BIU. As a result, the hypothesis H3 was formulated as:

#### **H3**

FC has an affirmative significant influence on students’ BIU towards using social networking tools for learning.

### Social influence SI

Social Influence refers to the process through which the students’ behavior, attitudes, and opinions are altered and amended by the acts/presence of other students. In numerous models for the reception of technology, Social Influence has emerged as the subject norm. Venkatesh et al. (2003) distinguish between mandatory versus un-mandatory situations since they discovered that social influence is important in the initial but not the later contexts. Extant studies on the impact of SI on technology adoption have also found similar results (Venkatesh et al., 2012). As a result, the following hypothesis was framed:

#### **H4**

SI has an affirmative significant influence on students’ BIU towards using social networking tools for learning.

### Intrinsic value IV

The present study incorporates the concept of social learning established by Albert Bandura, a Canadian psychologist, which emphasizes what an individual learns is influenced by the individual's physical and social surroundings. As a person learns by watching his parents, peers, and coworkers (Bandura, 1977), he also learns from the physical social environment. Using the same notion concerning Web 2.0 technology, he also learns from the virtual environment (Raspopovic et al., 2017). Researchers have proposed the concept of the expectancy-value framework of achievement motivation (Eccles et al., 1983), and value for subjective tasks (Chiu & Wang, 2008), for better understanding the students’ intent and for measuring the value’s influence on the utilization of technology.

Intrinsic Value as a concept is quite similar to intrinsic motivation since it entails getting a task done with inherent desire as well as pleasure (Wigfield & Eccles, 2000). IV includes two dimensions—interest and enjoyment. On the other hand, the hedonic motivation construct in the UTAUT-2 model is restricted to the dimension of enjoyment only (Venkatesh et al., 2012). The fun or amusement obtained from the utilization of any technology is termed hedonic motivation (Venkatesh et al., 2012). However, the IV variable is an addition of motivation, encompassing both pleasure and interest perspective, which has been used and modified by many of the researchers to suit their needs. Vanslambrouck et al. (2018) described IV as the enjoyment an individual derives from engagement or interest in education. Khechine et al. (2020) extended the UTAUT model by incorporating IV for studying the reception of a learning management system LMS, which integrated social learning tools. From the four variables that make up Subjective Task value: Expense and Values of Intrinsic, attainment, utility (Chiu & Wang, 2008), the extended model included only IV, as many of the aspects in the last three constructs were identical to those in the original UTAUT model such as performance expectancy and effort expectancy (Khechine et al., 2020). Based on the literature review, the following hypotheses were framed:

#### **H5A**

IV has an affirmative significant influence on students’ BIU towards using social networking tools for learning.

#### **H5B**

IV has an affirmative significant influence on students’ intent towards using social networking tools for learning.

### Behavioral intention for using learning tools BIU

Behavioral Intention denotes the students’ individualistic/subjective probability of performing certain behavior (Ajzen & Fishbein, 1980). Theoretically, the behavioral intention has a substantial affirmative impact on technology use. This connection was proven in several studies involving a variety of educational technologies. The following hypothesis was formulated:

#### **H6**

Behavioral Intention (BIU) has an affirmative significant influence on students’ intent towards using social networking tools for learning.

## Methodology

### Research design and participants

The design for research used for the present study is cross-sectional, employing a quantitative method to get responses from the students for assessing the students' intention for using social networking tools for learning. The data was gathered during the academic session 2020–2021 from the college students studying in an engineering institute in the State of Rajasthan, India. The study focused only on engineering students, as the age and level of experience of using tools may influence the students’ perceptions. Moreover, the stream of education, educational experience, and the background of the student can influence the findings; therefore, only engineering students were recruited for the study. The sample was chosen using the random sampling method, which gave all the students in the populace equal chances to contribute to the study, and of being selected for the sample; online random number generator was used to generate 350 numbers after each student was assigned a number from 1 to 1000. The selection depended entirely on probability. The method was chosen as it is a fair method for sampling and helps reduce biases. Moreover, it is one of the popular methods of sampling used in comparable studies. The questionnaire was circulated and 305 students responded, 48 females (15.7%) and 257 males (84.3%), with an average age of 18 years. The response rate was approximately 87%. All the students had been using social networking tools for learning for 1 year during the lockdown period. The social learning tools being used included YouTube, Wiki, Instagram, Facebook, Blogger, Google Meet, etc. The students were motivated to use these tools for sharing ideas, pictures, posts, audio/video files, etc. Using social networking tools, the interaction between students and the program/content/course was ensured. These tools were utilized for informing peers/parents/faculty about virtual/ real-world activities and events. They enabled the students to connect with others in diverse settings, and for varied activities.

### Data analysis

Partial least squares structural equation modeling (PLS-SEM), a methodology of structural equation modeling which allows the assessment of any complex cause-effect model (Hair et al., 2017), comprising latent variables was used for assessing the hypothesized model using SmartPLS version 3.2.9 (Ringle et al., 2015). It is a popular multivariate procedure that permits the consideration of flexible suppositions and provides an exact assessment of the hypothesized relationships (Garson, 2015, 2016).

### Instrument

The instrument used for gathering data for the empirical study consisted of 30 items. The questionnaire was divided into two sections: the first section comprised demographic, questions such as gender, age, etc., and the second section comprised items on UTAUT and Intrinsic values constructs. Prior validated scales were adopted for the current study. The statements were graded on Likert scales, from 1 to 5 demonstrating strongly disagree and strongly agree, respectively. Similar questionnaires have been extensively used for evaluating the reception of m-Learning among college students (Ameri et al., 2020; Baydas & Yilmaz, 2018; Kumar & Bervell, 2019).

### Common method bias/variance

Since both endogenous and exogenous factors have been gathered simultaneously utilizing a single instrument, Common Methods Bias (CMB) was used to guarantee that the data was not distorted. Two methodologies were used. Initially, utilizing the SPSS version 26, Harman's single-factor test investigation was done for all the constructs. The highest value observed was below the 50% level recommended, indicating the data did not have CMB. Nonetheless, as researchers disagree about employing Harman's single-factor investigation for CMB (Podsakoff et al., 2003, 2012), Pearson's Correlation Matrix was also checked to ensure there was no correlation above 0.90 that suggests the data may have CMB. All the correlations were lower than 0.80, which demonstrates the data does not have CMB.

### PLS-SEM

Smart PLS 3.2.9 software was used for partial least squares (PLS) and for assessing external estimations (reliability and validity) and internal structural model (validating hypotheses/direct and indirect effects) (Hair et al., 2017; Ringle et al., 2015). PLS was considered as the best option as Structural equation modeling is possible with modest sample sets in comparison to other available software. Moreover, PLS doesn't need a normally distributed data set, being a non-parametric methodology. Next, the study centers on predicting a model (relationships between select constructs and BIU/IU). Moreover, PLS-SEM is progressively gaining popularity in clarifying complex research problems (Henseler et al., 2016) and is utilized for enhancing the illustrative and explorative limit of main factors and their connections (Hair et al., 2014). As such, it was viewed as a suitable choice. The following sections of the paper describe the outcomes of the measurement and structural models (Hair et al., 2016; Marcoulides & Saunders, 2006).

## Results

### Measurement model—convergent validity (CV)

Tests were conducted to ensure the data is reliable and has satisfactory convergent validity. Table 1 shows the convergent validity findings are acceptable. To ensure the reliability of indicators is above 0.05 as the normalized loading above 0.708 (Hair et al., 2017), and substantial (*p* < 0.001), few items were deleted i.e. IU1, PE2, and SI4. Next, Cronbach's alpha for every factor was calculated, as indicated in Table 1, which was higher than 0.7. Besides, all Average Variance Extracted (AVE) values surpassed the verge of 0.50, showing the CV of the constructs was apposite for the model (Fornell & Larcker, 1981; Hair et al., 2012, 2017; Henseler, 2017; Henseler et al., 2016). Moreover, there were no issues with Composite Reliability, as Dijkstra-Henseler's rho (RhoA), the utmost significant condition for PLS (Dijkstra & Henseler, 2015), which gives a more exact assessment of data consistency was found to be adequate; the values of CR demonstrated that the constructs were valid and reliable (Ringle et al., 2017), as indicated in Table 1. Moreover, the inner Variance inflation factor VIF values were all below 5, which is the prescribed threshold.

**Table 1:** Convergent validity (Loadings, CA, CR, AVE, and VIF)

Items	Loadings	CA	rho_A	CR	AVE	VIF
BIU1	0.840	0.860	0.861	0.860	0.673	2.150
BIU3	0.823					2.619
BIU4	0.797					2.028
EE1	0.818	0.864	0.872	0.864	0.616	1.971
EE2	0.878					2.440
EE3	0.749					2.269
EE4	0.682					2.056
FC1	0.860	0.832	0.837	0.827	0.547	1.771
FC2	0.751					1.992
FC3	0.677					3.005
FC4	0.653					3.017
IU2	0.791	0.756	0.756	0.756	0.608	1.585
IU3	0.768					1.585
IV1	0.770	0.844	0.844	0.844	0.575	2.481
IV2	0.764					2.750
IV3	0.755					1.805
IV4	0.742					1.898
PE1	0.869	0.908	0.910	0.908	0.767	2.635
PE3	0.839					3.394
PE4	0.918					3.197
SI1	0.600					1.638
SI2	0.755					1.733
SI3	0.893	0.793	0.822	0.793	0.569	1.663

### Discriminant validity (DV)

DV of select constructs was determined through two tests. To start with, PLS-SEM was used, for the total bootstrapping, the findings indicate that the estimations of the correlations between the constructs which are all reflective are less than 0.90 (Hair et al., 2017; Henseler et al., 2015) or under 0.85 (Kline, 2016). To endorse discriminant validity, the square roots of AVE of a latent variable, as indicated by the diagonal estimations as shown in italics should be higher than the squared correlations between the latent variable and other constructs. However, it was found that the criterion was not fulfilled for BIU. To ensure the criterion was fulfilled one more item, namely BIU2 with loadings below 0.07 was deleted. In addition, the cross-loadings were also checked and found to be acceptable. Table 2 shows the results of Fornell and Larcker Criterion.

**Table 2:** Fornell and larcker criterion analysis

	BIU	EE	FC	IU	IV	PE	SI
BIU	*0.820*						
EE	0.759	*0.785*					
FC	0.482	0.562	*0.739*				
IU	0.495	0.622	0.403	*0.780*			
IV	0.528	0.680	0.578	0.606	*0.758*		
PE	0.687	0.724	0.515	0.588	0.553	*0.876*	
SI	0.499	0.451	0.389	0.228	0.405	0.406	*0.754*

The Heterotrait–Monotrait Test (Henseler et al., 2015; Nitzl, 2016) was used, which was considered as the benchmark for good performance for verifying the discriminant validity. The HTMT ratios indicate the mean heterotrait-heteromethod correlations in relation to the mean monotraitheteromethod correlation. The result is shown in Table 3 indicates that all values were below the threshold of 0.90, thereby establishing the model fulfills the requirements for discriminant validity.

**Table 3:** HTMT ratio

	BIU	EE	FC	IU	IV	PE	SI
BIU							
EE	0.758						
FC	0.476	0.554					
IU	0.496	0.617	0.395				
IV	0.527	0.684	0.569	0.606			
PE	0.686	0.724	0.517	0.587	0.553		
SI	0.495	0.446	0.384	0.226	0.407	0.405	

To attain the measurable outcomes for confirmation of hypotheses formulated, the structural model was investigated utilizing bootstrapping, with Smart PLS 3.2.9 (Ringle et al., 2015), to find statistical proof for assessing the accuracy of parameters. The outcomes display the model has prescient significance:

### Measures of R^2^ and Q^2^

A significant measure of the structural model assessment is an appraisal of the Coefficient of Determination i.e. R^2^. A limit estimation of 0.25, 0.5, and 0.7 as cutoffs depict weak, adequate, and strong results (Hair et al., 2014). Additionally, an appraisal of Stone-Geisser's predictive significance i.e. Q2 is essential to ensure Indicators’ data points in the measurement model of the endogenous variables are specifically anticipated.

Chin (1998) claims that predictive significance is confirmed when Q^2^ estimation is above zero, which indicates that the exogenous variables possess extrapolative predicting power for the endogenous variables being considered (Hair et al., 2014). As such, it is evident from Table 4, that the hypothesized model has great extrapolative significance for the endogenous factors.

**Table 4:** R^2^ and Q^2^ of the Endogenous Variables

	R^2^	R^2^ Adjusted	Q^2^ (= 1-SSE/SSO)
BIU	0.640	0.634	0.393
IU	0.410	0.406	0.214

### Model fit indices

Fit Indices such as Standardized Root Mean Square Residual (SRMR) and Normed Fit Index (NFI) were utilized to find the model fit (Henseler et al., 2014). SRMR calculated as the difference between the noticed connection and the anticipated connection is considered as an outright good fit measure sufficient for PLS-SEM-based models. The SRMR values below 0.1 or 0.09 confirm the PLS model’s fit (Hair et al., 2014, 2016). NFI values from zero and one are considered Good Fit; the value close to one is considered as a good fit (Ringle et al., 2017). The results of the model (saturated) as indicated in Table 5, show that SRMR value was 0.048 and the NRI value was 0.883. The results demonstrated that the model has a good fit and satisfies all criteria (Dijkstra & Henseler, 2015; Hair et al., 2016).

**Table 5:** Model fit indices

	Saturated model	Estimated model
SRMR	0.048	0.052
NFI	0.883	0.828

### Hypothesis testing

**Fig. 2:**
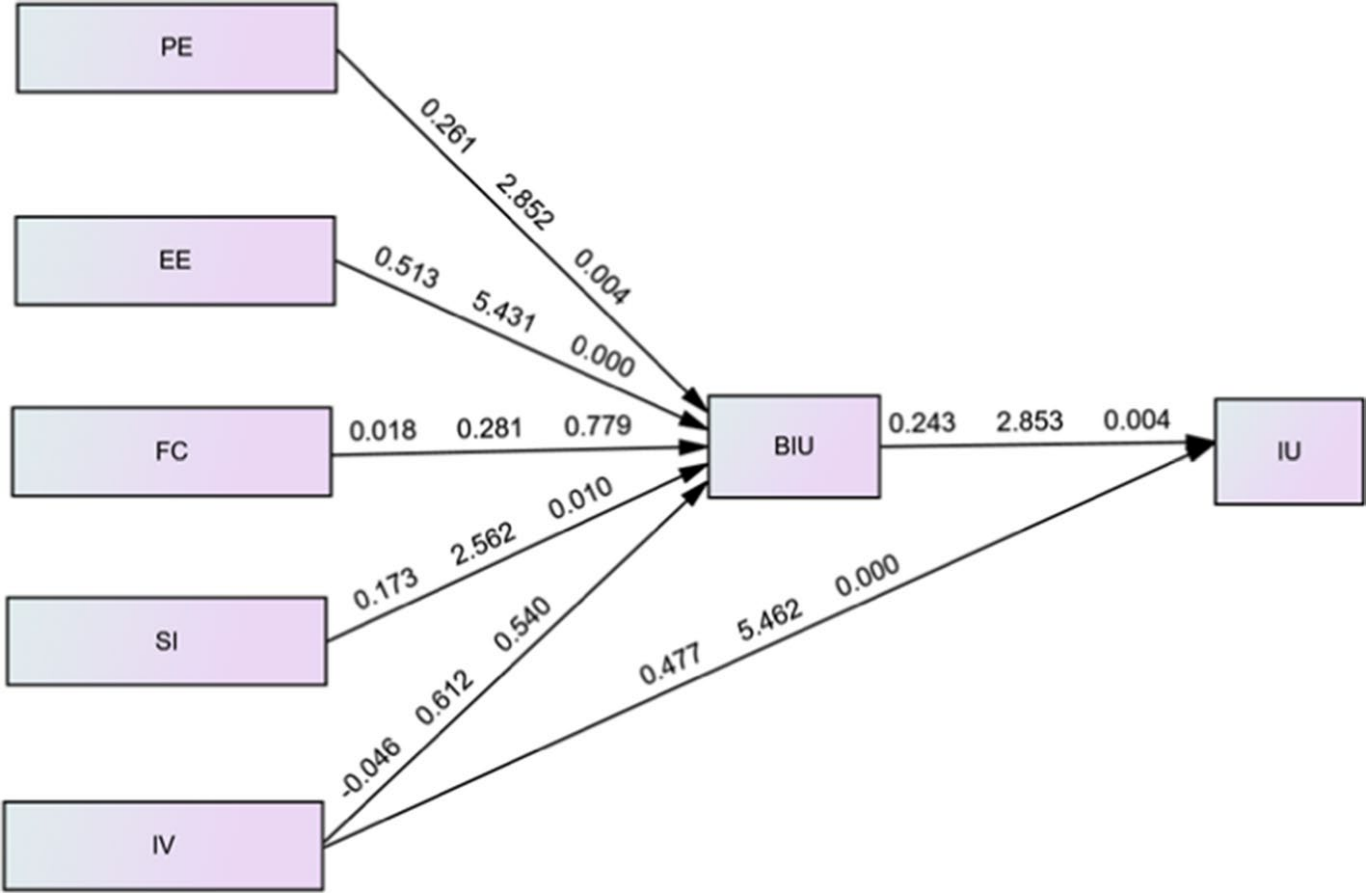
Research Model showing relationships between the select constructs and the β, t, and *p* values for each path

The hypotheses formulated for the study were verified by administrating the process of bootstrap with 5000 re-samplings (Hair et al., 2014). The findings are presented diagrammatically in Fig. 2.

All of the hypotheses formulated except H3: FC —> BIU and H5A = IV —> BIU were confirmed, as shown in Table 6. The hypothesized association between PE and BIU, as indicated by H1 was found to be meaningful and affirmative (β = 0.261, t = 2.852, *p* = 0.004). The basic characteristics of the education system during the COVID 19 situation can explain this outcome. Almost no classes could be conducted in any institute of Higher education, as attendance was impossible; before the pandemic, students thought the teacher's lecture in the classroom was more essential than the assigned course content; however, this was no longer the case. Due to the lockdown, the students had to depend on the content provided using social networking tools for learning. As a result, from the students' viewpoint, these tools were perceived as a significant contributor to academic success, since the learning tools offered the content which they needed. The findings affirm that college students recognize the fact that using these tools enables them to achieve academic goals. Many studies on the UTAUT model from 2004 to 2011 (Williams et al., 2015) corroborate the relationship between PE and BIU. More recent findings by researchers also support the results (Ahmed & Kabir, 2018; Kumar & Bervell, 2019).

**Table 6:** Summary of hypotheses testing

	β	STD	T Statistics	P Values	CI LL	CI UL	Results
H1: PE —> BIU	0.261	0.092	2.852	0.004	0.069	0.403	Accepted
H2: EE —> BIU	0.513	0.095	5.431	0.000	0.326	0.698	Accepted
H3: FC —> BIU	0.018	0.064	0.281	0.779	− 0.111	0.141	Rejected
H4: SI —> BIU	0.173	0.067	2.562	0.010	− 0.199	0.101	Accepted
H5A: IV —> BIU	− 0.046	0.076	0.612	0.540	0.309	0.651	Rejected
H5B: IV —> IU	0.477	0.087	5.462	0.000	0.089	0.451	Accepted
H6: BIU —> IU	0.243	0.085	2.853	0.004	0.04	0.306	Accepted

The relationship between EE and behavioral intention has a significant and affirmative path coefficient (β = 0.513, t = 5.431, *p* = 0.000); as such, Hypothesis H2 was accepted. Prior researches have also shown EE is a determining factor for intention towards utilizing novel technologies (Wang & Wang, 2010). For the current investigation, it can be interpreted that the students’ employment of social learning tools isn’t fraught with physical, intellectual, emotional, and psychological efforts, in other words, the social networking tools are easy to use for studying purposes.

The relationship between FC and behavioral intention was not significant (β = 0.018, t = 0.281, *p* = 0.779), demonstrating that H3 was unsupported. The existence of FC contributes to students' willingness for using social networking tools for learning. Venkatesh et al. (2012) established the noteworthy and affirmative association between FC and BIU, but the findings of the present study result differ from their findings. Although numerous researches have established facilitating conditions were a significant predictor of BIU in the context of the present study, FC was found to have no significant influence, indicating that the students did not consider the organizational, technological, and other pertinent tools, which are accessible for supporting the utilization of the social networking tools as very important. The findings differ from prior findings in the context of the adoption of new technologies for learning (Kim & Lee, 2020, etc.), which stresses the need for training, documentation, support systems, availability of trained for assisting the students in overcoming problems in utilizing the tools. The relationship between SI—> BIU has a significant and affirmative path coefficient (β = 0.173, t = 2.562, *p* = 0.010), demonstrating that H4 is validated. The present findings are in line with Moorthy et al.’s (2019) as well as Ameri et al.’s (2020) findings, endorsing that college students trust the insights of their parents, peers, and teachers, which consequently influence their own choices regarding social networking learning tools. Moreover, the students were further motivated towards using social networking tools for studies as they observed others' support for the same.

The influence of intrinsic value on behavioral intention was negligible (β = -0.046, t = 0.612, *p* = 0.540), while its influence on intent to use was affirmative and significant (β = 0.477, t = 5.462, *p* = 0.000), indicating that an affirmative view of intrinsic value among students may make them more likely to utilize the social learning system. Thus, H5A was rejected, but H5B was verified. Intrinsic Value is an important predictor of intent for learning using technology, according to Chiu and Wang (2008). It may be argued that providing students with the best possible environments for utilizing social networking tools when intrinsic values are unfavorable will prove to be fruitless. Furthermore, it should not be assumed that students who are used to these tools will inevitably experience affirmative IV from simply utilizing them for learning. Finally, the relationship between BIU and IU has a significant and affirmative path coefficient (β = 0.243, t- = 2.853, *p* = 0.004), which validated Hypothesis H6. The results established that BIU was a predictor of the student's intent to use these tools, which affirms the findings by Ameri et al. (2020), indicating that the students aspire to use them in the future, as well.

## Discussion

The objective of the present research was to find out what factors influence students' perspectives towards social networking tools for learning. An extended and modified proposed UTAUT model was introduced and evaluated using empirical data. The construct intrinsic value was included and evaluated for the adoption and successful utilization of social networking tools for learning, by checking essential motivators using the proposed model. The findings of the study helped to clarify the role of extrinsic and intrinsic motivators in the reception of social learning tools. They show PE, EE, and SI have a significant and affirmative relationship with BIU towards social networking tools for learning among students of Higher Education. The findings affirmed prior results obtained by García Botero et al. (2018), who confirmed that UTAUT constructs such as PE, FC, and SI swayed students’ outlooks towards technology-enabled learning in Higher Education, and those by Al-Adwan et al. (2018) who confirmed that EE, PE, and SI were important factors for acceptance of new technologies for learning. The findings are also corroborated by Onaolapo and Oyewole (2018), who showed that an affirmative association existed between PE, EE, and FC and acceptance of technological tools.

As such, the present findings support previous studies which testified to the acceptance of social tools as they support learners and reinforce learning (Al-Rahmi & Zeki, 2016). The ease in their availability, simple interface, and other user-friendly features add to their appeal (Benson et al., 2015). Moreover, the importance of encompassing the hedonic motivation variable by addition of the interest element to the enjoyment element, which contributes to the intrinsic variable, is one of the research's key theoretical contributions. Many prior studies (Ain et al., 2016), excluding a few such as Nikolopoulou et al. (2020) have demonstrated that hedonic motivation does not significantly impact behavioral intent based on students’ perspectives, which confirms the present findings. When Hedonic motivation was made more comprehensive by using the intrinsic value and its influence was found to be significant for intent to use. The findings show that the intrinsic value construct must be considered for innovations whose adoption is influenced by feelings of pleasure and interest in using social learning tools and tools for learning.

The results have several theoretical and practical implications. This study contributes substantial practical insight into the acceptance of social networking tools for learning. The findings provide a more nuanced understanding of the key factors influencing the use of social networking tools in higher education in developing countries such as India. It also provides useful recommendations to developers of social networking tools, policymakers, educational researchers, and teachers, empowering them to improve quality of social learning. The study indicates that acceptability of social networking tools for learning is contingent on not only the qualities of the tools described previously, such as PE, EE, and SI, but also on the intrinsic values as perceived by the students. Thus, administration should place a premium on these elements thereby contributing to students' acceptance of social learning, and enhancing students’ academic performance. Secondly, technical support departments must provide sufficient hardware, software, and technical help, as well as update resources constantly to enable the students to adopt social learning tools efficiently. Thirdly, the findings may help policymakers/ administration/ faculty to focus on strategies for boosting student awareness and knowledge about the social networking tools by organising training sessions/ courses to ensure that they are capable of effectively using the tools. The findings may be useful for designers/ developers of social networking tools for determining the students' requirements and priorities before creating the networking tools, hence avoiding post-implementation failures. Finally, the empirical findings from this study may assist stakeholders in making informed decisions about social networking tools acceptability, hence facilitating the successful implementation of new learning initiatives in the context of higher education.

## Conclusion and limitations

The main aim of the present research was to explore the factors influencing the students’ behavior towards the use of social networking tools for learning in higher education and evaluating the intrinsic and extrinsic motivators for their reception in India. The study also extended the scope of UTAUT to encompass the social networking tools for Higher Education. The findings provide substantial significance to the reception of social learning tools among college students. The theoretical impact of the research is the effort to augment technology acceptance scholarly writings by validating the UTAUT model in the context of the utilization of social networking tools for learning. The present outcomes offer a better insight into their implementation in India. This study provides a precedent by offering a statistically validated UTAUT model on a subject that is not covered in the existing literature: students’ acceptance of the social networking tools for learning. Another contribution of this research is the inclusion of IV as a construct. The fact that intrinsic value is a predictor of the individual's intent for utilizing social learning system from a practical point of view suggests that more attention needs to be paid to making the system more lucrative and rewarding for the students. It is necessary to reduce monotony and take advantage of the novel characteristics of social networking tools which the learners might feel as beneficial. Decision-makers should not only understand the functionalities of the social learning tools, but also focus on making them as entertaining, enjoyable, and motivating tools for learning. Institutes of higher education may foster positive attitudes about their utilization for education. Lastly, PE, EE, and SI may be improved to increase the acceptance of the social networking tools for learning.

The results indicate that the students in India have a constructive approach to the implementation of social networking tools for learning. Students believe they are useful, and are inclined towards using them. Nevertheless, to successfully integrate these tools in developing countries, several of the aspects mentioned above must be taken into consideration. As the knowledge of students' observation and reception are decisive for the execution of technological education plans, faculty and administrators may consider the study and the proposed model useful for learning more about students’ preferences.

Studies are still limited when it comes to research on the students’ inclination towards the use of social networking tools for learning. The study filled the research gap by analyzing the self-reported responses of the students. However, there is a possibility that the data may be skewed or biased, due to the means of collection and sampling adopted; the sample was non-experimental, and data was collected and assessed only one time. As individual perceptions are based on experience acquired by individuals (Venkatesh et al., 2003), repetitive measures will allow researches a deeper interpretation and insight into the factors while also providing discernment into any changes in students’ acceptance towards social learning over time. Furthermore, the data were obtained from only students studying in Higher education institutes, resulting in a fairly homogeneous population. As a result, it is important to learn how students from various backgrounds see social learning tools and tools for better insight and comprehension of their acceptance among students. Additional latent and moderating factors can be used in future studies, besides PE, EE, and FC and SI to improve and augment the illustrative competence of the present model.

## Data Availability

The data will be made available on request.

## Abbreviations

UTAUT: The unified theory of acceptance and use of technology
PE: Performance expectancy
EE: Effort expectancy
FC: Facilitating conditions
SI: Social influence
IV: Intrinsic value
BIU: Behavioral intention
IU: Intent to use
